# The importance of using routine laboratory tests in the diagnosis and prognosis of patients with coronavirus disease 2019: Shedding light on clinical laboratory data in COVID‐19

**DOI:** 10.1002/jcla.24713

**Published:** 2022-10-17

**Authors:** Ehsanollah Ghaznavi‐Rad, Mahmood Khosravi, Mohammad Sayyadi

**Affiliations:** ^1^ Department of Medical Laboratory Sciences, School of Allied Medical Sciences, Infectious Diseases Research Center (IDRC) Arak University of Medical Sciences Arak Iran

**Keywords:** biochemical markers, COVID‐19, hematological parameters, inflammation, prognosis

## Abstract

**Background:**

Novel coronavirus (COVID‐19) pandemic has become a global concern and requires early detection, isolation, and treatment. Our purpose is to find some beneficial information by analyzing the COVID‐19 laboratory data to provide guidance for clinical practice.

**Material and Methods:**

In this study, 174 patients with confirmed COVID‐19 infection were admitted. We evaluated the hematological and biochemical parameters in these patients and in 80 healthy individuals.

**Results:**

We found that there was significant difference between WBC, LYM, RBC, HB, and HCT parameters of patients and healthy counterparts (*p* < .001), though there was no remarkable change between NEU, MONO, PLT, and other characteristics of RBC values of patients and the control group (*p* ≥ .09). Among the evaluated biochemical parameters, the values of CK‐MB and LDH in the patient group were significantly different from the control group (*p* < .01), while other biochemical indicators were in the normal range.

**Conclusion:**

Several hematological and biochemistry parameters, in particular WBC, LYM, RBC, HB, HCT, CK‐MB, and LDH, could be beneficial supplementary approach for COVID‐19 infection evaluation to confirm risk stratification and effective management.

## Graphical abstract

Prognosis of COVID‐19 patients using important biochemical and hematological indicators.

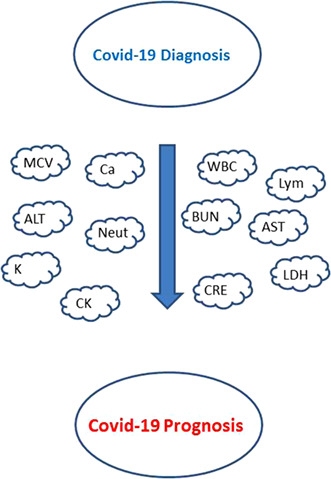

## 1 INTRODUCTION

Due to new emerging coronavirus disease 2019 with a very fast and progressive form, it is very crucial and vital to identify patients as soon as possible to decrease mortality rates and prevent severe and fatal conditions.^1^ For the first time, World Health Organization (WHO) called this disease caused by coronavirus as COVID‐19.^2^ Up to now, millions of infected people and more than 100 thousand deaths, which are rapidly increasing, have been reported worldwide.^3^ Although nearly 15% of the patients get complicated with the severe progress of the disease, large number of individuals who have positive RT‐PCR throat swab could be asymptomatic, and it is of concern to the governments to identify and differentiate COVID‐19 positive and negative patients to better control and prevent the spread of the disease.^4^, ^5^ The virus enters the body and starts to home and reproduce, then attacks some of the organs such as the pulmonary tract, gastrointestinal tract, and nervous system and can cause signs such as cough and fever; several laboratory analyses such as hematological and biochemical parameters can be used to identify the disease and separate into severe and nonsevere forms, which can lead to better manage the clinical outcomes.^6^, ^7^ Now with an increased number of reports and laboratory information, collecting some diagnostic and prognostic tests is very important to distinguish those who are at higher risk or lower risk of the disease.^8^ Up to now a lot of researches and reports have been published in international scientific journals worldwide and some reports have shown disease picture and been helpful to solve clinical problems of these patients.^9^ It is worth to mention that most studies aim to prepare more precise evaluation of the effect of laboratory tests in order to manage patients and improve clinical outcomes.^10^ On the other hand, due to the existence of false negative tests, which can be caused by the type of sampling or the type of test performed, the analysis of biochemical and hematological indicators seems necessary for the screening and rapid diagnosis of these patient.^11^, ^12^ Previous studies showed that several hematological values as hemoglobin (HB), hematocrit (HCT), white blood cells (WBC), platelets (PLT), and some others tend to alter in COVID‐2019 positive patients as well as some of biochemical features such as lactate alanine aminotransferase (ALT), aspartate aminotransferase (AST), and lactate dehydrogenase (LDH).^13^ Now as we know coronavirus has rapidly infected people around the world and cause a wide range of diseases from flu‐like to severe fatal forms in which about 20% of patients become critically involved, with a high mortality rate from 4.1% to 17%.^14^ In this study, we examined some laboratory characteristics such as hematological, biochemical, and liver tests in coronavirus‐affected patients to study the differences between severe and nonsevere forms and identify the importance of these parameters in recognizing and prognosis of COVID‐19 patients.

## 2 MATERIALS AND METHODS

### 2.1 Study population

We gathered the laboratory tests information from 174 patients diagnosed with COVID‐19 in the Amiralmomenin Hospital (Arak University of Medical Sciences, Iran) between June 1 and July 31, 2020 that comprised the patients group along with 80 healthy volunteers as control group. The diagnosis of COVID‐19 patients was according to World Health Organization guidelines^15^ and confirmed by RNA detection of the coronavirus in the clinical laboratory of Arak University of Medical Sciences. The control group was exposed to the same tests as the patients group, including clinical and laboratory inspection, chest x‐rays, and real‐time reverse transcriptase polymerase chain reaction (RT‐PCR) for COVID‐19, all tests consequence negative. The consent form was accepted by the research ethics committee at the Arak University of Medical Sciences (ID number: IR.ARAKMU.REC.1399.178) and informed consent was obtained from every contributor in agreement with the Declaration of Helsinki.

### 2.2 Sample collection and tests performed

We collected blood sample from both the healthy volunteers and the patients with COVID‐19 using conventional procedures and investigated hematological and biochemical values. The hematological parameters included white blood cell count (WBC), lymphocyte count (LYM), monocytes count (MONO), neutrophil count (NEU), red blood cell count (RBC), hemoglobin (HB), mean corpuscular volume (MCV), hematocrit (HCT), red blood cell volume distribution width‐CV (RDW‐CV), mean corpuscular hemoglobin (MCH), mean corpuscular‐hemoglobin concentration (MCHC), platelet count (PLT), and mean platelet volume (MPV). The biochemical parameters included serum alanine aminotransferase (ALT), serum aspartate amino transferase (AST), serum lactate dehydrogenase (LDH), serum potassium (K), calcium (Ca), serum creatinine (CRE), serum creatine kinase (CK), serum CK isoenzyme MB (CK‐Mb), serum uric acid (UA), blood urea nitrogen (BUN), and C‐reactive protein.

### 2.3 Statistical evaluation of the data

All quantities were stated as mean and standard deviation. The normality of the distribution was verified by single sample Kolmogorov–Smirnov test, followed by two‐tailed *t* test and Mann–Whitney *U* test that were used as appropriate to compare the continuous variables in the data from different patient groups. The variables are expressed as means and *p* values <.05 were considered statistically significant. spss version 23.0 was used for data evaluation.

## 3 RESULTS

### 3.1 Demographic characteristics of the patients

Among all the participants of the patients group, the mean age was ~46 years, 61% (106 of 174) were male and 39% (68 of 174) were female patients. In the control group, 70% (56 of 80) were male and 30% (24 of 80) were female participants. The age was not significantly changed between patients and control groups. The participants were divided into different subgroups including 95 individuals in the regular group, 53 participants in the severe group, and 31 patients in the critical group. There was no notable alteration in the median age and sex ratio of these subgroups. The most common clinical findings in all patients were fever, cough, and sputum production, and severe shortness of breath was seen more in severe and critical patients. The demographics and clinical features of patients with COVID‐19 are summarized in Table 1.

**TABLE 1:** Demographic and clinical characteristic of the patients

Study population	% of patient samples^a^
Sex	
Male	61
Female	39
Age (years)	
Median (32)	
Range (1–64)	
0–21	10
21–35	22
35–55	52
>55	16
ESR (mm/h)^b^	
Median (25.34)	
Range (1–90)	
0–10	21
10–20	36
20–30	24
>30	20
Symptoms	
Fever	81
Cough	72
Sputum production	71
Shortness of breath	54
Anorexia	63
Diarrhea	26
Fatigue	31
CRP (qualitative)	
Negative (4%)	
Positive (96%)	

Abbreviation: CRP; C‐reactive protein.

^a^ Percentages based on a total of 174 patients.

^b^ Erythrocyte sedimentation rate (mm/h).

### 3.2 Hematologic parameters of COVID‐19 patients and control group

The alterations of hematologic characteristics between patients with COVID‐19 and control group included the evaluation of values, as illustrated previously. Compared with healthy participants, we found that the white blood cell (WBC) count in patients with COVID‐19 were in the usual reference variety; nonetheless, the mean quantity in the severe and critical patients was higher than that in the regular group (*p* < .02 and *p* < .01, respectively). Lymphocyte count (LYM) was decreased in most patients, and lower in severe and critical COVID‐19 patients (*p* < .01). No relationship was detected between NEU and the disease status (*p* > .05), however, several patients had different degrees of RBC and decreased hemoglobin levels, and were more obvious in severe and critical patients (*p* < .05). Our results show that platelet count and MPV were generally normal in COVID‐19 patients. The results of the hematological findings are detailed in Table 2.

**TABLE 2:** Comparisons of hematologic parameters between patients and control group

Parameter	Control group	Total patients (*n* = 174)	*p*	Patients subgroup		
				Regular (*n* = 95)	Severe (*n* = 53)	Critical (*n* = 31)
WBC (×10^9^/L)	5.31 ± 2.34	6.94 ± 3.67	˃.05	6.41 ± 1.38	7.74 ± 2.51	8.45 ± 4.29
NEU (×10^9^/L)	4.83 ± 2.01	5.12 ± 1.98	˃.05	4.76 ± 1.54	5.10 ± 2.95	5.41 ± 2.41
LYM (×10^9^/L)	2.41 ± 0.48	1.32 ± 0.79	≤.05	1.43 ± 0.76	1.31 ± 0.98	1.12 ± 1.06
MON (×10^9^/L)	0.34 ± 0.15	0.37 ± 0.18	˃.05	0.36 ± 0.25	0.31 ± 0.21	0.32 ± 0.12
RBC (×10^12^/L)	5.62 ± 1.45	4.16 ± 1.89	˃.05	4.74 ± 2.01	4.32 ± 1.09	3.67 ± 0.97
HB (g/L)	15.64 ± 2.75	12.14 ± 2.41	≤.05	13.64 ± 1.19	13.02 ± 1.97	11.62 ± 2.97
HCT (%)	46.74 ± 6.84	35.81 ± 7.25	≤.05	39.02 ± 5.98	37.05 ± 5.74	34.71 ± 9.12
MCV (femtoliter)	86.25 ± 12.84	89.97 ± 10.26	˃.05	87.32 ± 10.15	89.64 ± 12.64	90.71 ± 7.46
RDW‐CV	11.12 ± 0.12	11.18 ± 0.81	˃.05	11.12 ± 0.61	11.30 ± 0.73	11.41 ± 0.94
MCH (picogram)	29.12 ± 2.77	28.91 ± 2.94	˃.05	29.05 ± 3.02	29.01 ± 3.15	28.62 ± 2.74
MCHC (g/L)	34.31 ± 1.18	35.01 ± 2.68	˃.05	34.91 ± 3.15	35.15 ± 2.97	34.99 ± 2.47
PLT (×109/L)	252.61 ± 42.61	198.21 ± 74.26	˃.05	224.18 ± 38.52	176.32 ± 29.63	189.29 ± 52.36
MPV (femtoliter)	8.32 ± 1.6	8.63 ± 1.02	˃.05	8.31 ± 0.97	8.47 ± 1.21	8.94 ± 1.14

### 3.3 Serum biochemical parameters of COVID‐19 patients and control group

The differences in serum biochemical parameters between COVID‐19 positive participants and healthy control group included the evaluation of factors, as illustrated earlier. Compared with control group, we found that the indicators K, Ca, BUN, CRE, UA, ALT, AST, CK, and CK‐Mb in patients with COVID‐19 were mostly within the reference range. However, LDH levels in COVID‐19 patients were significantly higher than the control group (*p* < .05). The results of the biochemical findings are detailed in Table 3.

**TABLE 3:** Comparisons of serum biochemical parameters between patients and control group

Parameter	Control group	Total patients (*n* = 174)	*p*	Patients subgroup		
				Regular (*n* = 95)	Severe (*n* = 53)	Critical (*n* = 31)
ALT (U/L)	34.26 ± 12.36	37 ± 10.87	˃.05	35.15 ± 11.19	39.84 ± 9.63	36.68 ± 10.24
AST (U/L)	26.15 ± 10.57	29.12 ± 22.84	˃.05	30.29 ± 23.82	29.79 ± 21.73	28.07 ± 22.15
CK (U/L)	36.01 ± 7.27	42 ± 9.43	˃.05	44 ± 8.61	41 ± 10.73	40.09 ± 8.96
CK‐Mb (U/L)	6.08 ± 3.87	8.21 ± 8.64	˃.05	7.12 ± 2.69	8.44 ± 3.98	9.32 ± 4.15
LDH (U/L)	115.78 ± 21.74	228.03 ± 31.86	≤.05	201 ± 38.91	221 ± 29.37	241 ± 25.76
K (mMol/L)	3.91 ± 0.25	3.74 ± 0.39	˃.05	3.64 ± 0.35	3.76 ± 0.42	3.91 ± 0.41
Ca (mMol/L)	2.33 ± 0.61	2.50 ± 0.41	˃.05	2.31 ± 0.53	2.67 ± 0.38	2.74 ± 0.42
UA (mMol/L)	0.25 ± 0.14	0.29 ± 0.74	˃.05	0.26 ± 0.68	0.29 ± 0.93	0.32 ± 0.78
BUN (mMol/L)	2.64 ± 0.36	2.77 ± 0.87	˃.05	2.81 ± 0.79	2.77 ± 0.86	2.63 ± 0.93
CRE (mMol/L)	75.27 ± 7.68	81.31 ± 10.25	˃.05	82.32 ± 11.36	80.76 ± 9.68	81.93 ± 9.94

## 4 DISCUSSION

There is a clear picture of the hematological, biochemical, and liver function tests derived from recent studies among patients with COVID‐19 unlike clinical symptoms that are the same as other respiratory syndromes.^16^, ^17^ COVID‐19 is one of the most important causes of respiratory syndrome which is of global concern and clinical symptoms include fever, fatigue, and cough that spread among people through human to human by different ways.^18^ Due to its high transmission risk and increased mortality rate, and other similar infections, laboratory analyses are essential for rapid diagnosis of patients in order to manage treatment.^19^ In our study, by focusing on hematological, biochemical, and liver function tests, we analyzed 174 patients comprised as the patients group along with 80 healthy volunteers as a control group. Our research showed no significant increase in WBC count in COVID‐19 patients compared to the healthy individuals; WBC count was notably increasing in patients with severe and critical status, but the LYM count in COVID‐19 patients was considerably reduced compared to the control group. Our research clarified that there is a significant relationship between the strength of COVID‐19 disease status and the increase in the WBC count, which is due to the increase in the number of neutrophils. Some study presented similar results to our study and stated that, as it has proven that lymphocytes are crucial to fight against viruses, and increase in most of viral infections and lead to lymphocytosis, conversely in COVID‐19 it tends to decrease.^20^, ^21^, ^22^ Platelet/lymphocyte ratio (PLR), neutrophil/lymphocyte ratio (NLR), and C‐reactive protein (CRP), three significant inflammatory indicators, were higher in COVID‐19 positive patients than control group.^23^ The cause of lymphocytopenia in COVID‐19 is not clear, but probably it is because of virus attraction or immune‐mediated lymphocyte apoptosis.^24^ Low levels of lymphocytes could be one of the possible causes of severe clinical symptoms of COVID‐19.^25^, ^26^ It is worth to note that, in our study no association was observed between NEU, MONO, and the disease status; additionally, we found that RBCs count (as in the study of Xiaohong Yuan et al.^27^) was lower in COVID‐19. Also, we found a decrease in hemoglobin level in COVID‐19 patients compared to control group. Our study show that several patients had different points of RBC, HB, and HCT decrease, and were more apparent in severe and critical patients compared to control group. Wang et al.^28^ showed that Hb and RDW might be a predictor of prognosis in severe COVID‐19 patients. Platelet counts and MPV unlike the study of Lippi et al.^29^ was generally in normal range in COVID‐19 patients. Bashash et al. reported that having established the link between PLT count and the outcome of the COVID‐19 patients, soon attention has been engrossed to other PLT‐related values and showed that MPV may also have a prediction probability in COVID‐19.^30^ The most interesting finding of our study is from liver function tests that contrary with Qingxian Cai results that ALT and AST were three times increased,^31^ in our study they were normal. Praveen Kumar‐M et al. demonstrated the obvious association between the levels of ALT and AST elevation and the severity of COVID‐19 patients' status,^32^ while in our study, there was no clear association. Serum biochemical parameters such as BUN, CRE, UA, K, Ca, CK, and CK‐Mb in COVID‐19 patients were mainly within the normal range. LDH levels were significantly higher in COVID‐9 patients like the study of Brandon Michael Henry et al.^33^ Although Khalid et al.^34^ reported that increased LDH values in COVID‐19 patients can be described by the simultaneous lactic acidosis, specifically present in malignant patients and subjects with comorbidities like heart disease, henceforth increasing related complications. As Guyi Wang et al.^35^ demonstrated an association between the risk of COVID‐19 aggravation and inflammatory marker CRP, we showed that CRP levels tend to increase in COVID‐19 patients too. There were some limitations in our study that should be noted, like we just enrolled patients to our study who had come to Amiralmomenin hospital and no other hospitals of Markazi province, and unknown clinical history of patients which affect our findings, hence more research is suggested.

## 5 CONCLUSIONS

By comparing hematological, biochemical, and liver functional parameters of 174 patients who were molecular confirmed, after being admitted to the hospital with COVID‐19 signs and symptoms, we found statistically noteworthy changes in the blood values of WBC, LYM, CRP, and LDH between those who were positive at the genetic test and healthy individuals. Consequently, not only a routine blood test might assist in identifying false RT‐PCR results, but also might be used in developing countries and in those states suffering from an unavailable RT‐PCR reagents or specialized tests to identify and manage patients with COVID‐19.

## CONFLICT OF INTEREST

The authors declare no potential conflicts of interest with respect to the research, authorship, and/or publication of this article.

## Data Availability

We agree to data availability statement.
